# Safety and Success of Lumbar Puncture in Young Infants: A Prospective Observational Study

**DOI:** 10.3389/fped.2021.692652

**Published:** 2021-06-14

**Authors:** Luca Bedetti, Licia Lugli, Lucia Marrozzini, Alessandro Baraldi, Federica Leone, Lorenza Baroni, Laura Lucaccioni, Cecilia Rossi, Maria F. Roversi, Roberto D'Amico, Lorenzo Iughetti, Alberto Berardi

**Affiliations:** ^1^PhD Program in Clinical and Experimental Medicine, University of Modena and Reggio Emilia, Modena, Italy; ^2^Neonatal Intensive Care Unit, Women's and Children's Health Department, University Hospital of Modena, Modena, Italy; ^3^Pediatrics, Women's, and Children's Health Department, University Hospital of Modena, Modena, Italy; ^4^Pediatric Post-graduate School, University Hospital of Modena and Reggio Emilia, Modena, Italy; ^5^Neonatal Intensive Care Unit, Santa Maria Nuova Hospital, Reggio Emilia, Italy; ^6^Unit of Statistics, Department of Diagnostic, Clinical and Public Health Medicine, University of Modena and Reggio Emilia, Modena, Italy

**Keywords:** lumbar puncture, meningitis, sepsis, newborn, pre-maturity, very low birth weight

## Abstract

**Objective:** This study aims to evaluate safety and success rates of lumbar puncture (LP) and to identify factors associated with adverse events or failure of LP in infants.

**Methods:** This two-center prospective observational study investigated infants younger than 90 days of age who underwent LP. Need for resuscitation oxygen desaturation (SpO_2_ < 90%), bradycardia and intraventricular hemorrhage were considered adverse events. LP failed if cerebrospinal spinal fluid was not collected or had traces of blood. Logistic regression analysis was used to evaluate whether corrected gestational age (GA), body weight at LP, position, and any respiratory support during LP affected SpO_2_ desaturation or failure of LP.

**Results:** Among 204 LPs, 134 were performed in full-term and 70 in pre-term born infants. SpO_2_ desaturations occurred during 45 (22.4%) LPs. At multivariate analysis, lower GA at LP (*p* < 0.001), non-invasive respiratory support (*p* 0.007) and mechanical ventilation (*p* 0.004) were associated with SpO_2_ desaturations. Transient, self-resolving bradycardia occurred in 7 (3.4%) infants. Two infants had intraventricular hemorrhage detected within 72 h of LP. No further adverse events were registered. Failure of LP occurred in 38.2% of cases and was not associated with any of the factors evaluated.

**Conclusions:** LP was safe in most infants. Body weight or GA at LP did not affect LP failure. These data are useful to clinicians, providing information on the safety of the procedure.

## Introduction

The gold standard for the diagnosis of bacterial meningitis is a positive cerebrospinal fluid (CSF) culture, usually obtained through a lumbar puncture (LP) (1). The clinical suspicion of meningitis is greater in the presence of seizures, fever, bulging fontanel and abnormal consciousness, but in neonatal age the initial signs are often subtle, particularly in younger infants (2). Guidelines from the United States of America and United Kingdom recommend performing an LP in cases of suspected sepsis and meningitis in neonates and infants (3, 4).

However, LP is probably performed less frequently than it should be according to the recommendations. Clinicians sometimes may be reluctant to perform an LP because they fear adverse events, they consider a LP technically difficult or excessively invasive. The likelihood of performing an LP seems reduce with decreasing birth weight (5–7). Indeed, a recent study from the United States reported that even in confirmed early-onset sepsis only ~80% of full-term infants and ~40% of very pre-term infants undergo LP (5).

There are two main concerns regarding LP in infants: the safety and the success rates of the procedure. Although these aspects have been extensively investigated in adulthood and in children (8, 9), limited data is available in neonates with a lower gestational age (10).

Thus far, safety has been mainly evaluated in terms of vital sign stability and the occurrence of SpO_2_ desaturation and bradycardia during an LP. Five prospective studies included sick infants with variable gestational ages (11–15). Most of them evaluated only a few pre-mature infants (12–15), and only one study (11) investigated a larger population of pre-mature infants; however, the risk of complications according to gestational age and birth weight remains poorly defined (8, 9, 16). Failure rates in young infants (age, 0–90 days) have been addressed in seven prospective studies; most of them included full-term neonates, and some included infants up to 1 year of age (8, 9, 17–21). Risks of failure of LP seem higher in infants younger than 90 days of age (8, 9). The present study aimed to assess safety and failure rates of LP.

## Materials and Methods

### Aim of the Study

The primary aim of the study was to evaluate adverse events associated with LP. As a secondary aim, we assessed the failure rates of LP.

### Study Design

This prospective observational study evaluated pre-term and full-term infants undergoing diagnostic LP in two tertiary level Italian hospitals (Azienda Policlinico, Modena; Azienda Santa Maria Nuova, Reggio Emilia). Infants younger than 90 days of age who were admitted to the Neonatal Intensive Care Units or to the Pediatric Departments between June 1, 2016 and December 31, 2019 were included. The study was approved by the local ethical committee (protocol 11/16), and written parental consent was obtained.

### Procedure

As per practice in our centers, LP was usually included in the sepsis workup, but it was deferred in case of severe sepsis/septic shock (usually requiring catecholamine support) or in case of instable mechanically ventilated neonates. LPs were performed by physicians or residents. However, the decision regarding who had to start the procedure was left to the physician's discretion, usually depending on the severity of the infant's disease. Any procedure started by residents was performed under the supervision of physicians, who were ready to intervene if residents failed or the newborns worsened.

During the procedure, a nurse flexed the infant's hip and spine, avoiding neck flexion. The decision regarding the newborn's position during LP (sitting or lateral recumbent, both at maximal hip flexion) was made at the clinician's discretion. The procedure could be performed in one or more attempts. An attempt was defined as any single penetration of the needle into the skin; the duration of the procedure was defined as the time between the insertion of the first needle and the extraction of the last one.

A 22-gauge needle was used, and the stylet was always in place while advancing the needle. According to our protocol, a local anesthetic (EMLA) was applied on the spinal skin 30–60 min before performing the LP. Intravenous (midazolam 100 ug/kg) or oral (diazepam 0.2 mg/kg) sedatives or pain relievers (fentanyl 1 ug/kg ev) were given at the clinician's discretion. LP was not performed if the infant had congenital malformations of the spinal region, bleeding disorders, or cellulitis at the site of LP.

Vital signs were evaluated during the time between insertion of the first needle and removal of the last needle. Monitors (IntelliVue MP40 Neonatal or IntelliVue MP2; Philips) were used for vital signs monitoring.

### Data Collection

A standardized form was *ad-hoc* developed for collecting the data. Information on infants undergoing LP was reported by a clinician involved in the procedure. The data form included patient-related factors (sex, gestational ages and body weights at birth and at LP, indication for LP, catecholamine or any respiratory support); who performed the LP (physician or resident); and factors related to the safety and success of LP (vital signs monitoring, need to start or increase oxygen or increase respiratory support during the procedure, need for resuscitation, patient position, number of attempts, the occurrence of any lesion at the skin site within 48 h after LP and evaluation of intraventricular hemorrhage).

Safety of LP was defined as the absence of adverse events. We defined as adverse events the need of intubation or cardiopulmonary resuscitation after LP; oxygen desaturation [oxygen saturation (SpO_2_) <90%] and transient bradycardia (heart rate <100 beats/min) were considered as secondary adverse events. We also evaluated the rate of intraventricular hemorrhage (IVH) assessed by ultrasound scans within 72 h of LP.

Failure of LP was defined as any of the following: inability to collect CSF or the occurrence of traumatic LP. An LP was considered traumatic if traces of blood could be seen at a visual threshold (22) or if the CSF was bloody.

### Statistical Analyses

The characteristics of infants, clinicians, and procedure were summarized using descriptive statistics. Continuous variables were expressed as mean ± standard deviation or median and interquartile range, whereas categorical variables were expressed as frequencies. Adverse events or failure of LP and factors associated with both (i.e., position, who performed the LP, corrected gestational age, body weight at LP and respiratory support) were estimated at uni- and multi-variate logistic regression analysis. We used forward selection strategy for including variables in multivariable model. A *p* valued < 0.05 was considered significant. The odds ratio was used as a measure of association, and it was reported with its 95% confidence interval.

We estimated that a sample size of 220 LPs provided a two-sided 95% CI with a width equal to 0.13, when the sample proportion of LP failure is around 0.35 (35%). This proportion was estimated on the basis of previous published studies on this topic (17,18,20). Because no substantial information was available regarding SpO_2_ desaturations during LP, the sample size was estimated based on failure rates.

## Results

### Characteristics of the Population and Procedure

Among 204 LPs, 153 were performed in infants (75%) admitted to the neonatal intensive care units and 51 (25%) were performed in infants admitted to the pediatric ward (Tables 1, 2); 134 (65.7%) out of 204 were performed in full-term and 70 (34.3%) in pre-term infants. Forty LPs were performed in infants with a body weight <1,500 g at LP, of which 19 had a body weight <1,000 g.

**Table 1:** Gestational age and body weight at birth and at the time of lumbar puncture.

	**Median**	**IQR**	**Range**
GA at birth, weeks	39	31.5–40	24–42
GA at LP, weeks	40	36–43	25–50
Body weight at birth, g	3,000	1,480–3,500	498–4,700
Body weight at LP, g	3,260	1,907–3,953	524–7,600

*GA, gestational age; IQR, interquartile range; LP, lumbar puncture*.

**Table 2:** Respiratory support, position during lumbar puncture and additional information on performing lumbar puncture.

	**Lumbar punctures** ***N* = 204**
**No respiratory support**, ***n*** **(%)**^†^	84 (41.2%)
**Respiratory support during LP**, ***n*** **(%)**
- Low or high-flow nasal cannula, nCPAP^‡^	88 (43.1%)
- Mechanical ventilation^¶^	32 (15.7%)
**Position during LP**, ***n*** **(%)**^¥^
- Sitting only	121 (61.4%)
- Lateral recumbent only	76 (38.6%)
**Duration of LP, median minutes (IQR)**	5 (3–0)
**LP failure**, ***n*** **(%)**	78 (38.2%)

*IQR, interquartile range; LP, lumbar puncture; nCPAP, nasal continuous positive airway pressure*.

† *One infant had body weight at LP <1,500 g*.

‡ *Sixteen infants had body weight at LP <1,500 g*.

¶ *Twenty-three infants had body weight at LP <1,500 g*.

¥ *Seven lumbar punctures were performed with multiple attempts both in sitting and lateral recumbent positions. Rates are calculated on the remaining 197 LPs performed in sitting or lateral recumbent position only*.

Among 204 LPs, 108 were performed only by physicians and 96 by residents. LPs were more commonly performed by physicians alone in infants on mechanical ventilation (22 of 32 LPs) or those with a body weight <1,500 g at the time of LP (29 of 40 LPs).

Regarding the position 7 LPs were attempted in both sitting and lateral recumbent position. Among the remaining 197 LPs with a single position, sitting was the preferred position; however, all but 1 LPs in infants on mechanical ventilation (*N* = 32) were performed by placing the infants only in the lateral recumbent position only.

### Safety of Lumbar Puncture

#### Cardiopulmonary Instability

No infants required intubation or cardiopulmonary resuscitation after LP.

Seven full-term infants received supplemental oxygen prior to LP in order to prevent SpO_2_ desaturation. No infants who were breathing in room air were given oxygen during LP. Oxygen was increased during LP in 13 infants (body weight <1,500 g, *N* = 8) who were on non-invasive respiratory support. Oxygen or inspiratory pressure were increased during LP in 10 of 32 (31.3%) infants on mechanical ventilation (body weight <1,500 g, *N* = 8).

**Figure 1:**
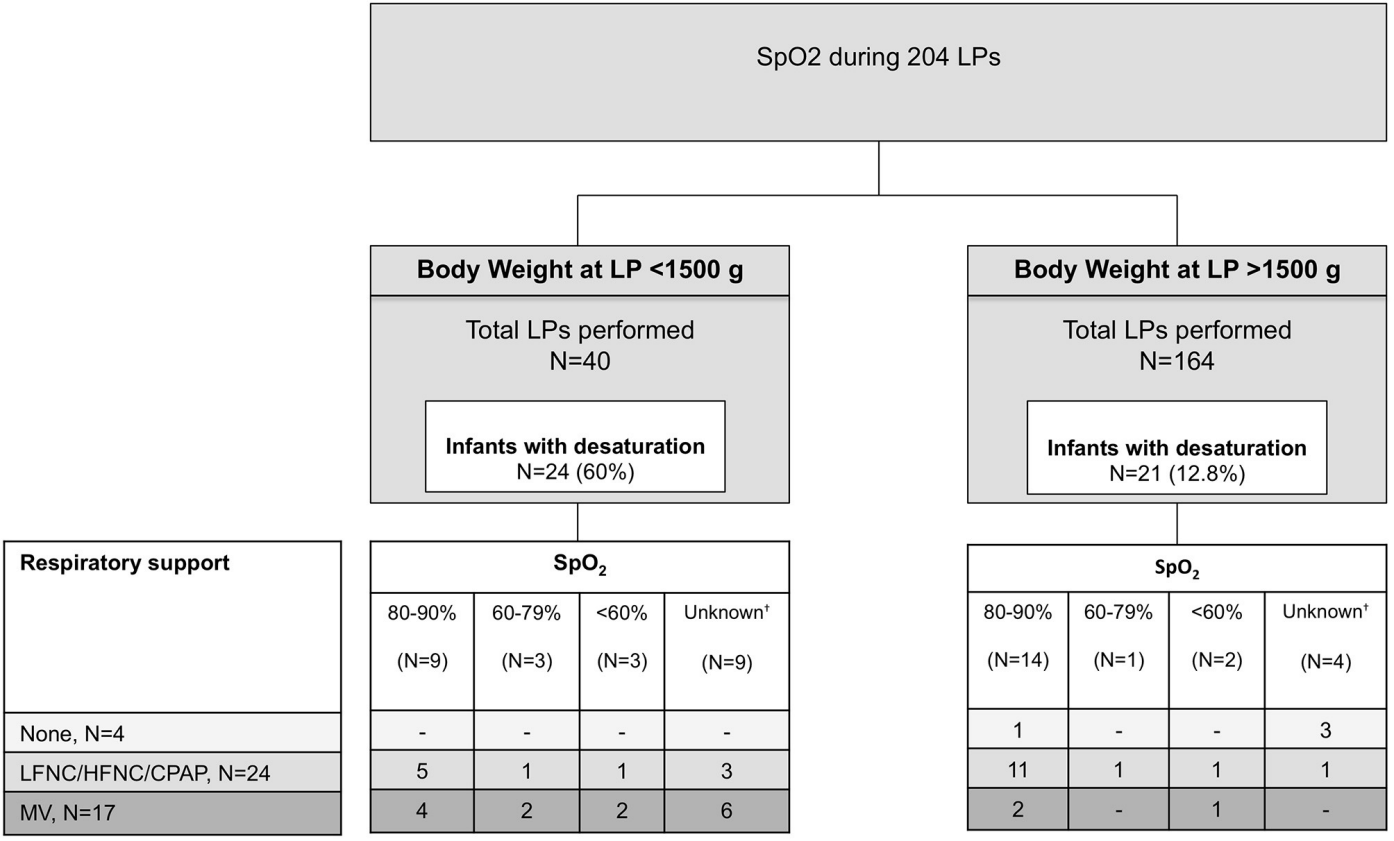
SpO_2_ desaturations (<90%) in 204 infants who had vital signs assessment during LP. SpO2 desaturations are divided according to their severity. HFNC, high-flow nasal cannula; MV, mechanical ventilation; LFNC, low-flow nasal cannula; LP, lumbar puncture; nCPAP, nasal continuous positive airway pressure.^†^Cases in which the standardized form indicates “desaturation” but without reporting the degree. Twenty of 204 (10%) of LPs were performed in infants on catecholamine support (dobutamine, dopamine or both). Among 20 LPs, 11 were performed in infants with body weight <1,500 g at the time of LP; 8 infants were on CPAP and 12 on mechanical ventilation. Desaturations occurred in 9 infants on catecholamine support.

Figure 1 shows the rates of SpO_2_ desaturations (SpO_2_ <90%) during 204 LPs performed in infants who had vital signs assessment. Forty-five LPs (22.1%) were performed in infants who had transient SpO_2_ desaturations during LP, although the value of SpO_2_ desaturation was not specified in 13 cases. SpO_2_ desaturations were significantly more common when LP was performed in infants with a lower body weight at LP (24 of 40 with a body weight <1,500 g vs. 21 of 164 with a body weight >1,500 g, *p* <0.001). Only five infants had SpO_2_ desaturation <60% during LP. The footnote of Figure 1 reports infants on catecholamine support and the percentage of SpO_2_ desaturations in these infants.

Lateral position, lower gestational age and body weight (<1,500 g) at LP and any respiratory support were significantly associated with SpO_2_ desaturation at univariate analysis (Table 3); at multivariate analysis, risks of SpO_2_ desaturations remained associated with lower gestational age at LP and use of any respiratory support. No differences were found when LP was performed by physicians alone or by residents.

**Table 3:** Univariate and multivariate analyses of factors associated with SpO_2_ desaturations during lumbar puncture.

	**Univariate**			**Multivariate**		
	**OR**	**95% CI**	***p***	**OR**	**95% CI**	***p***
**Position**						
- Sitting only	1	–	–			
- Lateral recumbent only	2.00	1.13–3.55	0.016			
**Gestational age at LP, weeks**	0.84	0.79–0.89	<0.001	0.88	0.82–0.95	<0.001
**Body weight at LP, g**						
- ≥1,500	1	–	–			
- <1,500	10.21	4.67–22.30	<0.001			
**Respiratory support during LP**						
- Self-ventilating in room air	1	–	–	1	–	–
- Low or high-flow nasal cannula/nCPAP	7.49	2.47–22.72	<0.001	4.84	1.53–15.26	0.007
- Mechanical ventilation	22.66	6.68–76.84	<0.001	7.54	1.88–30.14	0.004

*LP, lumbar puncture; nCPAP, nasal continuous positive airway pressure*.

Transient, self-resolving bradycardia (heart rate over 70 beats/min) was observed during 7 LPs.

#### Further Complications

Among infants with body weight at LP <1,500 g, one had bilateral grade 1 IVH detected 24 h after LP and one had unilateral grade 2 IVH detected 72 h after LP, when a severe septic shock was diagnosed. No local complications at the LP site or further adverse events were observed.

### Failure of Lumbar Puncture

The median of attempts performed was 1 (IQR 1–2, range 1–6). Among 204 LPs, 78 (38.2%) were unsuccessful: they were traumatic (mildly bloody: *N* = 47) or CSF was not obtained (*N* = 31). The remaining 126 (61.8%) were successful (Table 2); most of them (85, 67.5%) required only one attempt. At univariate analysis we did not find associations with LP failure (Table 4).

**Table 4:** Univariate analysis of factors associated with lumbar puncture failure.

	**Univariate**		
**Variables**	**OR**	**95% CI**	***p***
**Position**			
- Sitting only	1	–	–
- Lateral recumbent only	1.05	0.58–1.92	0.853
**Gestational age at LP, weeks**	0.96	0.91–1.00	0.094
**Body weight at LP, g**			
≥1,500	1	–	–
<1,500	1.03	0.50–2.12	0.915
**Respiratory support during LP**			
- Self-ventilating in room air	1	–	–
- Low or high-flow nasal cannula/nCPAP	0.84	0.45–1.55	0.597
- Mechanical ventilation	0.88	0.38–2.03	0.770
**Who performed LP**			
- Physician	1	–	–
- Resident ± Physician	1.55	0.88–2.74	0.127

*LP, lumbar puncture; nCPAP, nasal continuous positive airway pressure*.

## Discussion

This study aims to evaluate safety and success rates of LP in sick young infants aged 0 to 90 days. Our data shows that most LPs are safe, while the success rate is ~60%.

This is the largest prospective study investigating these aspects in infants; it includes about 20% of pre-term infants, who have the highest risk of meningitis (23). Adverse events and failure of LP have been evaluated according to different gestational ages and body weight, and information is provided in real-life settings, as suggested by others (24).

Since LP plays a pivotal role in diagnosing meningitis, clinicians should know if LP is associated with risks and, if so, the extent of the risks.

No infants required intubation after LP, cardiopulmonary resuscitation or drugs. Positive inspiratory pressure was increased after LP in a few infants who were mechanically ventilated.

In literature, the safety of LP in young infants has been mostly evaluated according to the severity of SpO_2_ desaturations (11, 13, 14). Unfortunately, these studies only concern a few newborns, and do not allow us to understand the true extent of the SpO_2_ desaturation. In order to better assess the actual risks, some have mimicked LP by positioning sick or healthy infants in different positions (24–26). These studies have shown that the lateral recumbent position is risky, particularly after maximal hip flexion (i.e., the “knee-chest” position). We found a strong association between the lateral recumbent position and SpO_2_ desaturations at univariate analysis; however, since all but one infant on mechanical ventilation were placed in the lateral recumbent position, we could not confirm this association by means of multivariate analysis.

It is unclear whether desaturations are related to the LP itself or sometimes to the positioning of the infants during the procedure (11, 26). Furthermore, during mechanical ventilation additional unintended risks may occur, since it is possible that the endotracheal tube dislocates. Therefore, we recommend the utmost care in preparing the infant to LP, paying attention to the correct position of the endotracheal tube. To date, only one study, which includes 7 pre-term and 9 full-term infants, has investigated the risks of SpO_2_ desaturation when LP is performed in neonates who suffer from respiratory disease (14). That study has found that the risk is significantly higher in pre-term neonates with respiratory distress syndrome. Consistent with that study, we found that the highest risk of SpO_2_ desaturation occurred in infants under mechanical ventilation.

Furthermore, we found that SpO_2_ desaturations were more common in infants with a lower gestational age at the time of LP. We emphasize the importance of using special care in the pre-term infant undergoing LP. Nevertheless, only a few infants (being all on respiratory support) experienced severe SpO_2_ desaturations or transient and self-resolving bradycardia. No infants had evidence of severe complications. Both infants who had intraventricular hemorrhage, had additional risks for bleeding (septic shock and low body weight), independently from the LP.

Approximately 38% of LPs failed, a rate that is consistent with the 23% to 41% reported in three studies regarding infants younger than 90 days of age (17, 18, 20). LP failure was not affected by a lower corrected gestational age and body weight at LP; these findings reassure clinicians who are less confident with LP in younger infants. Failure rates were not affected by the position, nor by the clinician who performed the LP (physicians or residents); these results support the training of young doctors in performing LP (17, 18, 20, 22). However, the failure rate is particularly high and additional techniques [i.e., ultrasound assisted LP (19)] will probably improve success rates in the future.

The present study has some potential limitations. Firstly, we were unable to calculate a sample size for the primary aim, since studies on the safety of LP are lacking. Secondly, an unquantified number of LPs were deferred, being a potential bias for the calculation of adverse events. However, this number seems low (<5–10% of total LPs), since we usually defer an LP only in septic shock (usually requiring catecholamine support) or in the case of instable, mechanically ventilated neonates. Additionally, the definition of traumatic LP was based only on visual assessment without obtaining a CSF red cell blood count. Further factors [such as inability to palpate the spinous process (8, 9) and the use of sedation or anesthetics] that were not investigated in this study could have affected the clinical worsening of neonates or the risk of an LP failure. Finally, SpO_2_ levels were not reported in all cases of desaturation, although missing values were only a few of the total LPs.

In conclusion, this study shows that LP was safe in most infants younger than 90 days of age. The risk of SpO_2_ desaturations was higher in infants with a lower gestational age at LP or those undergoing mechanical ventilation. However, only a few infants required escalating respiratory support, and none was intubated or resuscitated after LP. Pre-maturity was not associated with an increased risk of LP failure. By means of this information clinicians will be more confident while performing an LP, especially knowing which infants have the greatest risks associated with the procedure.

## Data Availability Statement

The raw data supporting the conclusions of this article will be made available by the authors, without undue reservation.

## Ethics Statement

The studies involving human participants were reviewed and approved by Comitato Etico dell'Area Vasta Emilia Nord (Protocol 11/16). Written informed consent to participate in this study was provided by the participants' legal guardian/next of kin.

## Author Contributions

LBe and ABe conceptualized and designed the study, coordinated and supervised data collection, drafted the initial manuscript, and reviewed and revised the manuscript. LM, ABa, FL, and LBa collected data and drafted the initial manuscript. LLug, LLuc, CR, MR, RD'A, and LI critically analyzed and interpreted data, and critically reviewed the manuscript. All authors approved the final manuscript as submitted and agree to be accountable for all aspects of the work.

## Conflict of Interest

The authors declare that the research was conducted in the absence of any commercial or financial relationships that could be construed as a potential conflict of interest.
